# Ambient *operando* self-healing in tin perovskite solar cells

**DOI:** 10.1039/d5el00182j

**Published:** 2026-01-21

**Authors:** Miriam Minguez-Avellan, Omar E. Solis, Noemi Farinós-Navajas, Pablo F. Betancur, Jorge Pascual, Teresa S. Ripolles, Rafael Abargues, Pablo P. Boix

**Affiliations:** a Instituto de Ciencia de los Materiales-Universidad de Valencia Catedrático José Beltrán, 2 València 46980 Spain rafael.abargues@uv.es; b Instituto de Tecnología Química, Universitat Politècnica València-Consejo Superior de Investigaciones Científicas Av. dels Tarongers 46022 València Spain Pablo.P.Boix@itq.upv.es; c Polymat, University of the Basque Country UPV/EHU Donostia-San Sebastian 20018 Spain

## Abstract

Tin-based perovskite photovoltaics offer promising optoelectronic properties and a less toxic alternative to lead-based counterparts but suffer from rapid degradation under ambient conditions, primarily due to Sn^2+^ oxidation. We leverage the chemically dynamic nature of halide perovskites to demonstrate a functional *operando* self-healing effect in unencapsulated Sn-based perovskite solar cells (Sn-PSCs). This is enabled by incorporating thiophene-2-ethylammonium (TEA) halide as an additive in FASnI_3_ devices. Remarkably, under continuous ambient operation (30 °C, 60% RH) and one sun simulated illumination, these devices can spontaneously overcome initial performance losses, progressively enhancing their power conversion efficiency (PCE) beyond their initial values. In particular, following self healing, devices with TEAI retain 80% of their initial PCE for over 25 hours, whereas control devices degrade in less than one hour. This self-healing behavior is significantly influenced by external parameters such as illumination intensity or additional applied bias, key factors triggering or hindering the recovery process. The underlying mechanism is discussed in the context of the potential TEAI additive reducing capability. These unprecedented findings provide new insights into the dynamic stability and recovery behaviors of Sn-PSCs, a step toward stable and efficient lead-free perovskite solar cells. A deeper understanding of this phenomenon can be key to designing strategies for more sustainable photovoltaic technologies.

## Abstract 2

Tin-based perovskite photovoltaics offer promising optoelectronic properties and a less toxic alternative to lead-based counterparts but suffer from rapid degradation under ambient conditions, primarily due to Sn^2+^ oxidation.

## Graphical abstract

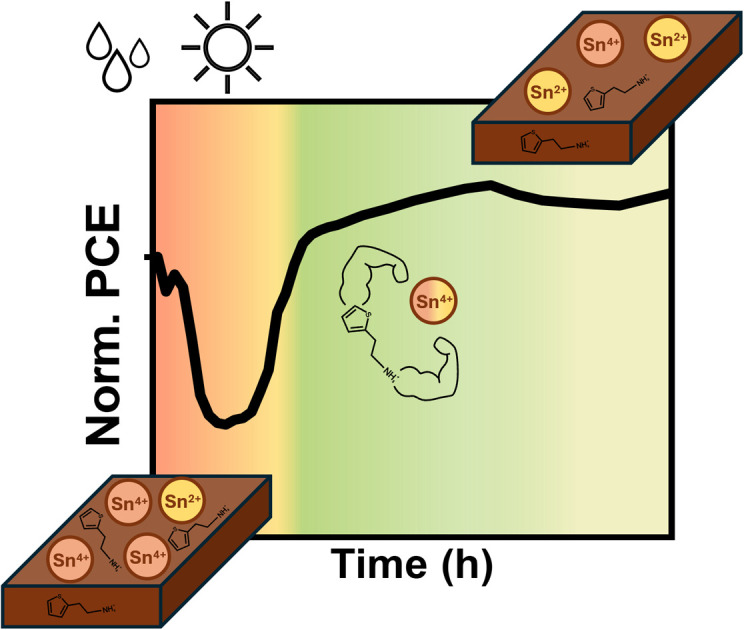

**Broader context**

A key limitation of perovskite solar cells is their poor stability when exposed to realistic operating conditions. Tin-based perovskites are an attractive lead-free alternative, but they suffer from rapid degradation due to the oxidation of Sn^2+^. This work addresses those challenges by introducing 2-thiopheneethylammonium iodide (TEAI) as an additive, which enables *operando* self-healing in tin perovskite solar cells. Devices containing TEAI spontaneously recover from initial efficiency losses during operation surpassing their original performance. Comprehensive electrical and optical characterization reveals that this effect originates from reversible chemical restoration processes, identifying the reduction of Sn^4+^ back to Sn^2+^ as the central mechanism. Understanding this dynamic redox-driven recovery provides valuable insight into how interfacial recombination and degradation pathways can be mitigated. This knowledge establishes a foundation for the rational design of additives and interfaces that enhance the stability and efficiency of tin-based perovskite solar cells, accelerating their development as viable lead-free photovoltaic technologies.

## Introduction

Halide perovskite semiconductors have gained significant attention in optoelectronics due to their exceptional properties, making them promising candidates for solar cells, photodetectors, and LEDs.^1–3^ Among them, lead-based perovskite solar cells have achieved remarkable progress in just 14 years, with power conversion efficiency (PCE) rising from 3.8% ^4^ to record values that reach over 27%.^5^ However, Pb toxicity remains a key concern, driving research into alternative materials.^6^

Tin-based perovskites have emerged as a viable lead-free alternative due to their similar electronic configuration (ns^2^ np^2^), which enables comparable optoelectronic properties, such as high charge carrier mobility and strong light absorption.^7,8^ Despite these advantages, tin-based perovskites solar cells (Sn-PSCs) suffer from lower device stability due to their faster crystallization and the facile oxidation of Sn^2+^ to Sn^4+^, especially under ambient conditions.^9–11^ This process generates Sn^2+^ vacancies, leading to excessive p-doping and charge trap states that degrade device performance.^12–14^

Various strategies have been explored to mitigate this instability and prevent Sn^2+^ oxidation,^15,16^ as well as enhance film quality, including the use of tin halides (SnCl_2_, SnF_2_)^17^ or gallic acid.^18^ The incorporation of bulky ammonium cations, commonly used in 2D perovskites, has also been demonstrated as an effective strategy to improve stability in Sn-based perovskite by improving the crystallization as well as providing hydrophobic protection.^19^ Among these, we recently proved that 2-thiopheneethylammonium halides (TEAX) show particularly promising potential due to their dual role in defect passivation and crystal lattice stabilization,^20^ achieving devices with >2000 h continuous performance under one sun-equivalent in N_2_ atmosphere. However, it is unclear if the dominating degradation mechanism can be fully circumvented. Moreover, despite the advances in encapsulation methods, a certain degree of resistance to ambient exposure (particularly to O_2_ and H_2_O), is essential for the practical development of Sn-PSCs.

Interestingly, the chemically dynamic nature of halide perovskites presents new opportunities to tackle this challenge. Various studies have reported the ability of Pb-halide perovskites to heal or repair themselves^21,22,23^ spontaneously and reversibly, both at a material level and at device level.^24^ While such effects have been much less studied in Sn-based perovskites, controlled environmental and light exposure, as well as the incorporation of specific additives, have shown potential for partial or even complete material recovery.^25,26^

In the present work, we report, for the first time, a pronounced self-healing effect observed in unencapsulated Sn-PSCs operating under ambient conditions. Remarkably, this phenomenon occurs spontaneously under continuous operational stress, without the need to interrupt the degradation stimulus, making it distinct from previously reported recovery mechanisms.^26^ The incorporation of 2-thiopheneethylammonium iodide (TEAI) significantly enhances the operational stability of the solar cells, extending their performance for over 20 hours under ambient conditions (30 °C and 60% RH) increasing their PCE about a 20% relative to their initial value and reaching a T80’ (time to achieve the 80% of the initial PCE) of ∼25 hours. Understanding this unique self-healing capability provides critical insight into the underlying degradation and recovery mechanisms, offering a promising route to significantly enhance the stability and durability of Sn-PSCs.

## Results

The synthesis and preparation of the Sn-PSCs, with a p-i-n configuration (ITO/PEDOT:PSS/Sn-perovskite/C_60_/BCP/Ag), follow a previously reported^20^ experimental procedure (see Experimental section for more information). The incorporation of a small amount (10 mol%) of thiophene-2-ethylammonium halides (TEAX, where X = I, Br, Cl) into the FASnI_3_ (FASI) precursor solution improves crystallization and reduces Sn^2+^ oxidation. As a result, the incorporation of TEAI salts enhances the PCE of the devices from ∼5% to more than 10% for champion devices, primarily through a significant improvement in open-circuit voltage (*V*_OC_), as detailed in Tables S1 and S2 and in Fig. S3a. The external quantum efficiency (EQE) measurements show that the integrated short-circuit current density (*J*_SC_) exhibits only a marginal increase upon TEAI addition, confirming that improved current extraction is not the main driver of performance enhancement (Fig. S3b). More interestingly, the devices exhibit improved stability, retaining over 95% of their initial PCE after 2000 hours of operation under continuous illumination in N_2_ conditions.^20^ This remarkable stability positions these devices as promising candidates for further testing in more demanding ambient environments. Although the best-performing devices reached PCE close to 11%, the following analysis is centered on solar cells with representative efficiencies of about 8%. This way, a more statistically consistent performance is studied, and it allows a clearer evaluation of the mechanism. Hereafter we focus on TEAI-containing devices to avoid additional complexities related to bandgap variations that occur with different halide substitutions. Nonetheless, equivalent behavior is consistently observed across all devices regardless of the TEAX halide composition (Fig. S1 and Table S3).

### Performance evolution under ambient conditions

**Fig. 1:**
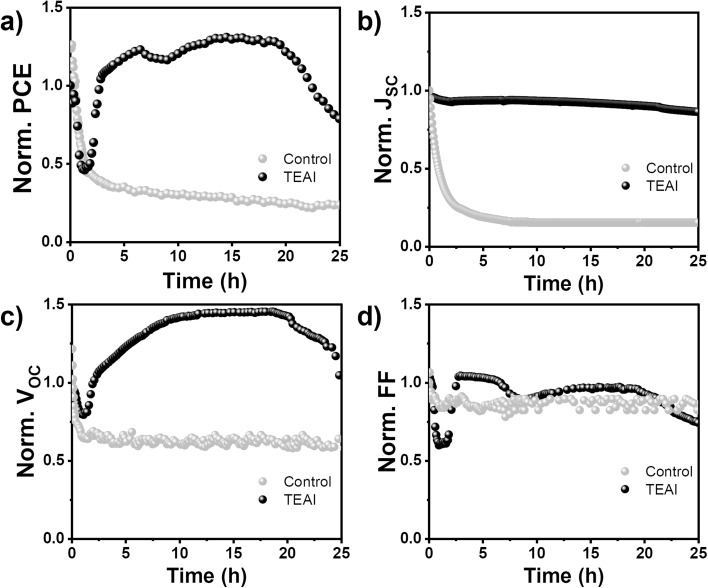
(a–d) Time evolution of solar cells normalized parameters of pristine FASnI_3_ (control) and FASnI_3_-TEAI (TEAI) measured under operation conditions of MPP and 1 sun illumination (100 mW cm^−2^) at ambient conditions of 30 °C and 60% RH.

Under ambient air and continuous illumination conditions, using maximum power point (MPP) bias and sequential current–voltage (*jV*) curves, Sn-PSCs typically exhibit degradation behavior characterized by rapid and irreversible performance decline.^9^ The pristine FASI PSC (without TEAI) demonstrates such an expected degradation pattern, showing a continuous drop in PCE over time, Fig. 1a. Such strong instability under ambient conditions is characteristic of unencapsulated FASI devices and is consistently observed for similarly processed devices.^20^ This performance deterioration is accompanied by concurrent decreases in *J*_SC_, *V*_OC_, and fill factor (FF), all following similar decline trends throughout the operational period, Fig. 1b–d. The observed reduction in *J*_SC_ is indicative of extraction or transport issues as Sn^4+^ concentration increases due to oxidation processes.^10^

In contrast to this conventional degradation behavior, Sn-PSCs incorporating TEAI additive exhibit an unprecedented self-healing phenomenon in Sn-PSC literature. Remarkably, these devices demonstrate complete recovery of their initial performance following an initial degradation phase during stability testing under identical operational conditions. Importantly, this self-healing behavior is fully reproducible across multiple TEAI-containing devices, as shown in Fig. S2. This anomalous performance evolution represents a fundamental divergence from expected PSC behavior and demands detailed investigation to understand the underlying mechanisms.

The self-healing effect observed in TEAI-containing Sn-PSCs manifests through a distinct temporal evolution of photovoltaic parameters during, mainly, the first 8 hours of operation under simulated 1 sun illumination and MPP bias. While the device initially experiences a decrease in PCE during the first hour of operation, this degradation is completely reversed after 3 hours, reaching a full recovery of the initial performance (Fig. 1a). Notably, the evolution of photovoltaic parameters reveals that TEAI devices exhibit a fundamentally different degradation mechanism compared to pristine FASI cells. While *J*_SC_ remains consistently stable in TEAI-containing devices throughout the entire operational period, *V*_OC_ and FF emerge as the dominant factors governing PCE variation. This behavior contrasts sharply with the pristine FASI PSCs, where *J*_SC_ degradation plays a key role in the performance decline (Fig. 1b).

The stable *J*_SC_ in TEAI devices, combined with the recovery of *V*_OC_ and FF (Fig. 1c and d), suggest that changes in recombination dynamics are the primary factors controlling performance variation. Thus, the TEAI additive fundamentally alters the recombination processes within the device, introducing additional dynamics that not only influence initial device stability but also enable the remarkable self-healing phenomenon under operational conditions.

Understanding these mechanisms is therefore a key objective to exploit the potential of the recovery process. The self-healing phenomenon exhibits a critical dependence on environmental conditions. While this unusual behavior is observed under ambient air conditions, it remains indiscernible when devices are tested in an inert N_2_ atmosphere, Fig. S3c. As previously reported, TEAI-containing Sn-PSCs characterized in a N_2_-filled glovebox (GB), remain remarkably stable for over 2000 hours.^20^ Thus, the self-healing effect only becomes noticeable when the Sn-perovskite material is exposed to degradation pathways, which are more abundant under ambient conditions. Under inert conditions, the absence of atmospheric oxidants and moisture prevents the initial degradation that would normally trigger the compensatory healing response.

Building upon the environmental requirements, the specific operational conditions under which devices are tested further modulate the self-healing behavior. Systematic variation of these parameters reveals the complex interplay between environmental exposure and operational stress in governing device behavior.

**Fig. 2:**
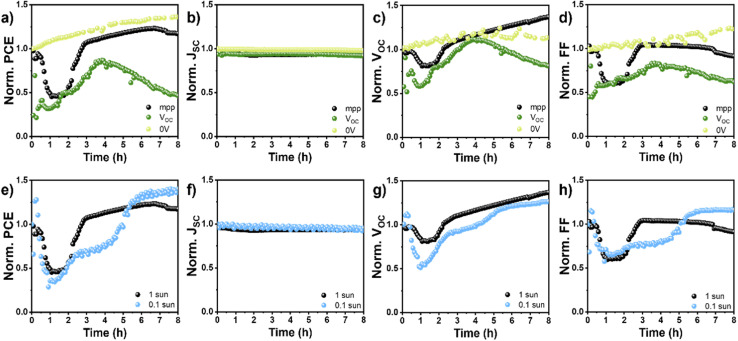
(a–d) Time evolution of FASI-TEAI solar cell normalized parameters measured under 1 sun illumination (100 mW cm^−2^) at ambient conditions of 30 °C and 60% RH at operation conditions of MPP (black), *V*_OC_ (dark green) and short-circuit conditions of 0 V (light green). (e–h) Time evolution of FASI-TEAI solar cell normalized parameters measured under operation conditions of MPP at ambient conditions of 30 °C and 60% RH at 1 sun illumination (100 mW cm^−2^, black dots) and 0.1 sun (10 mW cm^−2^, blue dots).

If the device performance is tested in similar ambient by sequential *jV*s scans, with the device held at *V*_OC_ conditions between those, TEAI-containing PSCs exhibit modified self-healing dynamics compared to sequential *jV*s scans with the device held at MPP conditions (Fig. 2a). The initial PCE drop occurs more rapidly, followed by an extended recovery period of approximately 4 hours, after which irreversible decline ensues. This behavior can be attributed to the combined effects of ambient exposure and altered charge carrier dynamics. At *V*_OC_, high photogenerated carrier densities accumulate without extraction pathways, leading to enhanced interfacial charge accumulation^27^ that, when coupled with ambient-induced degradation, intensifies both recombination losses and interfacial degradation processes.

Conversely, maintaining the devices at short-circuit conditions (0 V bias) between the *jV* scans in ambient air effectively suppresses the characteristic degradation–recovery cycle. Under these conditions, all photovoltaic parameters, PCE, *J*_SC_, *V*_OC_ and FF remain stable with slight improvement over time, Fig. 2a–d. The rapid extraction of photogenerated carriers at short-circuit condition minimizes interfacial charge accumulation, reducing the operational stress that, in combination with ambient exposure, typically triggers the initial degradation phase.

The intensity of the illumination provides another critical parameter governing the self-healing kinetics in ambient conditions. When devices are subjected to reduced illumination (0.1 sun) in ambient air, the recovery process becomes significantly slower, Fig. 2e–h. Lower illumination levels generate fewer charge carriers, thereby slowing down the processes responsible for the recovery. These observations highlight the importance of both electrical and optical excitation in driving the dynamic healing behaviour in Sn-based PSCs.

**Fig. 3:**
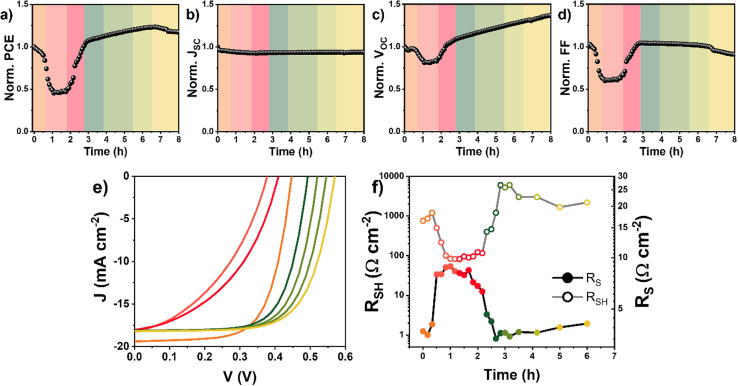
(a–d) Time evolution of FASI-TEAI solar cell normalized parameters measured under operation conditions of MPP and 1 sun illumination (100 mW cm^−2^) at ambient conditions of 30 °C and 60% RH. (e) *jV* characteristics and (f) series (*R*_S_) and shunt (*R*_SH_) resistances of the same solar cell at different stages of the self-healing process. Zone I: orange color represents initial values (0 h). Zone II: red colours represent low efficiency stages (1–3 h). Zone III: green (3–6 h) and zone IV: yellow (>6 h) represent the recovered solar cell stages.

A deeper analysis of the FASI-TEAI solar cell performance is necessary to extract comprehensive information about the mechanisms underlying the self-healing process. To facilitate this detailed investigation for understanding the temporal evolution of device behavior, the self-healing phenomenon has been divided into distinct operational time zones, each represented by different colors in Fig. 3. The complete self-healing cycle can be categorized into four distinct phases: zone I (orange, 0 h) represents the initial fresh state of the FASI-TEAI solar cell. Zone II (red, 1–3 h) encompasses the initial degradation phase and the onset of recovery. Zone III (green, 3–6 h) corresponds to the fully recovered state, where the device has regained its initial performance levels and demonstrates stable operation. Finally, zone IV (yellow, >6 h) represents the final operational state, providing insights into the long-term behavior of the self-healing cycle.

As previously mentioned, during the first hour of operation, a notable decrease in the PCE of the PSC containing TEAI is observed, driven by reductions in *V*_OC_ and FF as evidenced by the *jV* curves displayed in Fig. 3e. This initial degradation, caused by the oxidation of Sn^2+^ at ambient conditions, is correlated with a significant increase in series resistance (*R*_S_) and a minor decrease in shunt resistance (*R*_SH_), Fig. 3f. The rise in *R*_s_ suggests the generation of interfacial issues between layers within the device that hinder proper charge extraction,^28^ affecting mostly FF and PCE, Fig. 3a and d.

After approximately one hour of continuous operation, zone II of the process, the TEAI-device performance stagnates, with a stabilized PCE driven by *V*_OC_ and FF. This is reflected by the *R*_S_ and *R*_SH_, which also reduce their change rate, probably related to improvements in charge recombination and transport respectively.

After the second hour of continuous operation, the device performance experiences a new change, increasing significantly. This performance improvement is correlated with a progressive decrease of the *R*_S_, Fig. 3f. Simultaneously, *R*_SH_ increases beyond its initial values, suggesting improvement in material quality and reduced current leakage pathways. This increase in *R*_SH_ directly correlates with the recovery of *V*_OC_ and FF, as higher values of *R*_SH_ minimize parasitic current losses and improves device's rectification characteristics, Fig. 3c–e.

The increase in the *V*_OC_ beyond the third hour of continuous operation, zones III and IV, suggests that recombination rate continues to decrease (Fig. 3c). However, FF stabilizes in zone III and even decreases when entering zone IV (Fig. 3d). Since recombination appears to be further suppressed during zones III and IV, as indicated by the *V*_OC_ trend, the decline in FF is likely associated with charge transport limitations rather than recombination effects. This interpretation is supported by the gradual increase in *R*_S_ and the slight decrease in *R*_SH_ observed in zones III and IV (Fig. 3f).

### Mechanistic origin of the self-healing process

**Fig. 4:**
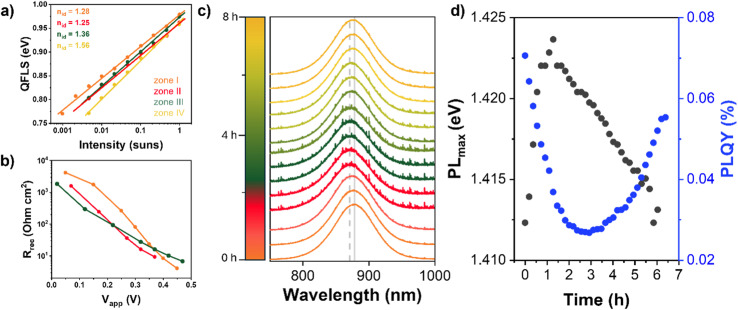
(a) Intensity dependent quasi-Fermi level splitting (QFLS), (b) recombination resistance (*R*_rec_) under 1 sun illumination as a function of the applied voltage (*V*_app_) and (c) PL intensity of a FASI-TEAI solar cell at the different stages of the self-healing process. Orange color represents zone I; red colours represent zone II; green colour (zone III) and yellow colour, zone IV. Solid gray line marks the position of the PL maximum (878 nm) for the zone I spectra (orange), and dash gray line marks the position of the maximum (870 nm) for the zone II spectra (red). (d) PLQY and energy variation of the PL maximum of the FASI-TEAI solar cell during the self-healing process.

Intensity-dependent photoluminescence (PL) measurements were carried out to obtain the quasi-Fermi level splitting (QFLS) of the FASI-TEAI devices along the self-healing process. The ideality factor, extracted from the QFLS dependence on light intensity, provides in-depth information on the recombination mechanisms.^29,30^ The evolution of these values calculated for the TEAI-containing Sn-PSC at each self-healing zone (Fig. 4a) offers compelling evidence that supports and refines the interpretations of the underlying physical mechanisms. The values closer to 1 for the device in zones I and II of the self-healing process suggest that direct recombination is governing, most likely occurring at interfaces.^29,31^ These findings corroborate the above-mentioned observations of *V*_OC_ and FF degradation during these phases, as interfacial recombination directly impacts these performance parameters. However, following the recovery process, zones III and IV, a notable increase in the ideality factor is observed, suggesting a fundamental shift in the dominant recombination mechanism toward trap-mediated processes, presumably bulk-dominated recombination pathways.^29^

To gain deeper insight into the evolution of charge carrier dynamics throughout the self-healing process, *operando* impedance spectroscopy (IS) measurements were performed under ambient conditions and one sun illumination on devices during distinct operational zones. Fig. S4a–c presents the Nyquist plots for these different stages under the same applied DC voltage. Under certain conditions, the impedance spectra display the conventional two-arc structure typically observed in PSCs. However, in most cases, the Nyquist plots are dominated by a single arc, which, at lower frequencies, can extend into the second quadrant (as previously reported for Sn-PSCs^32^). The recombination resistance (*R*_rec_), a parameter that directly reflects the recombination rate, can be unambiguously extracted by reconstructing the corresponding experimental *jV* curves (Fig. S4d–f) using a previously developed method.^33^ The evolution of the *R*_rec_, Fig. 4b, corroborates that the initial PCE drop in zone II is related to increased charge recombination. Interestingly, when entering zone III, charge recombination decreases again, as there is an increase in *R*_rec_. Interestingly, a notable change in the slope of the *R*_rec_*vs. V*_app_ is observed between these zones of the self-healing process, as shown in Fig. 4b, suggesting a change in the dominant recombination mechanism, in agreement with the ideality factor observations.

*Operando* PL and photoluminescence quantum yield (PLQY) throughout the self-healing process (see Experimental section for detailed methodology) provide direct information about the radiative and non-radiative recombination pathways governing the evolution of device performance. When moving from zone I to zone II of the self-healing process, a progressive blue shift of approximately 8 nm (12.5 meV) in the emission peak is observed (Fig. 4c and d), with this shift toward higher energies being previously linked to Sn^2+^ oxidation in FASnI_3_ perovskite systems.^34^,^35^ This spectral shift represents a clear fingerprint of Sn^2+^ oxidation occurring within the perovskite lattice during the initial degradation phase. Concurrently, the PLQY exhibits a systematic decrease (Fig. 4d), directly confirming the non-radiative nature of the enhanced recombination pathways identified through impedance analysis. This dual approach, combining electrical and optical characterization, confirms the increased recombination losses during the initial degradation phase. Critically, the PL spectra indicates that this oxidation process is reversible: upon entering zones III and IV of the self-healing process, the emission peak gradually red-shifts back toward its original position, indicating the progressive reduction of Sn^4+^ back to Sn^2+^. Consistently, devices with higher initial efficiencies present softer initial degradation and a faster overall recovery, (as displayed in Fig. S5 for a representative case) demonstrating that the self-healing phenomenon is also observed in well-performing devices and linked to Sn^4+^ content.

Following comprehensive electrical, impedance, and optical characterization, the results collectively confirm that the self-healing effect is driven by active chemical restoration processes. Thus, to confirm if the redox mechanism is indeed the foundation of device recovery, it is crucial to examine the specific reducing capabilities of TEAI using UV-vis spectroscopy and nuclear magnetic resonance (NMR) spectroscopy. Solutions of Sn(ii) + I^−^ and Sn(iv) + I^−^, were prepared in a DMF:DMSO mixed solvent. In solution, Sn(ii) reacts with I^−^ to form the [SnI_4_]^2−^ complex and Sn(iv) reacts forming a brownish solution of [SnI_6_]^2−^ (Fig. S6a), which exhibits two characteristic absorbance peaks at 290 and 365 nm (Fig. S6b). Upon addition of TEAI to the [SnI_6_]^2−^ solution, these absorbance peaks completely disappear in a matter of seconds (see video S1), indicating efficient reduction of Sn(iv) back to Sn(ii).^10^,^36^ This chemical reduction was further corroborated by ^119^Sn NMR spectroscopy (Fig. S6c). Solutions with [SnI_4_]^2−^ and [SnI_6_]^2−^ exhibit characteristic ^119^Sn NMR chemical shifts at ∼670 and ∼2020 ppm, respectively.^37^ Upon addition of TEAI, the [SnI_6_]^2−^ + TEAI solution showed no detectable resonance near 2000 ppm, indicating the disappearance of Sn(iv) species. Instead, a clear signal emerged at ∼668 ppm, which is attributed to the chemical shift of [SnI_4_]^2−^, thus confirming the complete reduction of Sn(iv) to Sn(ii).

Importantly, control experiments using other long-chain aromatic cations such as phenylethylammonium iodide (PEAI), 4-fluorophenylethylammonium iodide (FPEAI), as well as the short-chain ethylenediammonium diiodide (EDAI_2_), conducted under identical conditions, did not lead to any observable reduction of Sn(iv) to Sn(ii). In all cases, the characteristic absorbance bands at 290 and 350 nm remained after the addition of the salts, indicating the persistence of Sn(iv) species (Fig. S7). This clearly demonstrates that the reduction of Sn(iv) to Sn(ii) is uniquely enabled by the thiophene-based TEAI. These findings strongly support the conclusion that redox activity arises specifically from the electron-rich thiophene ring,^38,39^ and not from general aromaticity or ammonium functionality alone (Fig. S7). Therefore, we propose that the thiophene moiety may act as a reducing agent capable of mitigating oxidizing species present in the system, which could contribute to the enhanced stability observed experimentally. Two main hypotheses are considered to explain this behavior. A first plausible scenario involves redox-driven dimerization of thiophene units, a process reported in the presence of strong oxidants and which could, in principle, reduce species such as Sn(iv) or I_3_^−^. A second possible pathway is the oxidation of the sulfur center, leading to sulfoxide or sulfone derivatives, with ambient oxygen or other oxidizing agents acting as reactants. Moreover, oxidized thiophene analogues are known to undergo subsequent spontaneous coupling reactions, including Diels–Alder-type processes.^40^ These two hypotheses highlight the chemical complexity of the system and the existence of multiple competitive reaction pathways and will be the subject of further investigation, although they are not directly demonstrated by the current dataset. A complete mechanistic elucidation of TEAI chemistry, however, is beyond the scope of this work, which primarily focuses on the experimentally observed self-healing behavior and is currently under further investigation.

Comprehensive characterization through multiple complementary techniques provides a coherent picture of the self-healing mechanism. The systematic evolution of *R*_S_ and *R*_SH_ demonstrates the electrical manifestation of the recovery process, with *R*_S_ decreasing and *R*_SH_ increasing when entering zone III (Fig. 3f). Critically, this electrical recovery pattern aligns with the shift in ideality factors from values near unity to higher values, revealing a consistent narrative: as recovery takes place, recombination pathways redirect from direct-dominated recombination, probably at the interfaces, to trap-dominated mechanisms, at the bulk. This transition is reflected in both the improved resistance characteristics (lower *R*_S_, higher *R*_SH_) and the evolution toward higher ideality factors typical of bulk recombination processes.^29^ Impedance spectroscopy corroborates these findings through the evolution of *R*_rec_, directly linking electrical improvements to reduced recombination losses. Most critically, the PL evidence, featuring a reversible 8 nm blue-shift and concurrent PLQY recovery, provides unambiguous proof that the self-healing phenomenon stems from the chemical reduction of Sn^4+^ back to the Sn^2+^ species. The results obtained from the UV-vis absorption and ^119^Sn NMR spectroscopy of [SnI_4_]^2−^ solutions can be considered a proof-of-concept demonstration of TEAI's ability to reduce Sn^4+^ to Sn^2+^ species. It should be emphasized, however, that these experiments were carried out in solution. In the solid state, the environment within polycrystalline films under electrical bias and illumination is considerably much more complex. Factors such as grain boundaries, interfacial layers, restricted ion diffusion, and local electric fields can significantly influence the redox processes. Nevertheless, previous studies have shown that TEAI is homogeneously distributed throughout the perovskite film thickness,^20^ supporting its ability to interact effectively with Sn sites across the entire active layer. However, stability measurements reveal that this self-healing mechanism has limitations. Although devices maintain nearly constant performance for extended periods, continued exposure to light and ambient atmosphere eventually causes irreversible degradation. Notably, the *V*_OC_ returns to initial values after 25 hours of operation (Fig. 1c), while the FF progressively decreases after 6 hours, Fig. 1d. These trends in *V*_OC_ and FF together with the concurrent increase in *R*_S_ and slight decrease in *R*_SH_ (Fig. 3f) at later stages of the self-healing process, suggest the emergence of transport limitations. Given the relatively low TEAI concentration compared to the bulk perovskite, it is reasonable to assume that its ability to compensate the progressive formation of Sn^4+^ and other long-lived defects is eventually finite, which explains the final performance decay observed in long-term stability tests. At the same time, the possible contribution of additional degradation pathways, including the formation and accumulation of oxidized species or other by-products cannot be excluded.

## Conclusions

TEAI incorporation in Sn-based perovskite solar cells results in a unique *operando* self-healing phenomenon in unencapsulated devices. Under continuous illumination and MPP bias in ambient conditions, the devices recover and improve their initial performance, achieving a PCE enhancement of up to 20% and retaining 80% of its initial PCE for ∼25 h. This behavior contrasts with previously reported self-healing processes that typically require removal of stress stimuli. Through comprehensive electrical, optical and chemical characterizations, we demonstrate that the self-healing mechanism operates through active chemical restoration *via* mitigation of interfacial recombination processes. PL measurements provide direct evidence of chemical restoration through reversible spectral shifts and PLQY recovery, while solution-based studies confirm TEAI's ability to chemically reduce Sn^4+^ species. Our spectroscopic analysis establishes that the reductive ability of TEAI plays a central role in reversing Sn^4+^ formation, with both light intensity and electrical bias critical in activating the healing process. These findings open a new pathway for improving the operational stability of lead-free perovskite solar cells and establish the molecular foundation for designing chemically adaptive photovoltaic materials that can autonomously counter degradation mechanisms during operation.

## Conflicts of interest

There are no conflicts to declare.

## Supplementary Material

EL-002-D5EL00182J-s001

EL-002-D5EL00182J-s002

## Data Availability

All data supporting the results of this study is available at Zenodo at https://doi.org/10.5281/zenodo.18347072. Supplementary information (SI) is available. Supplementary information: experimental details (materials and methods), additional *J*–*V*, EQE, stability and impedance spectroscopy data, and tables summarizing photovoltaic parameters of control and TEAX-treated FASnI_3_ solar cells. See DOI: https://doi.org/10.1039/d5el00182j.
