# Supplementary material for: Ambient operando self-healing in tin perovskite solar cells
Source: EES Solar. 2026 Jan 21;2(2):360–8. doi: 10.1039/d5el00182j (PMC12848990; doi:10.1039/d5el00182j)
Supplement: EL-002-D5EL00182J-s001 [file EL-002-D5EL00182J-s001.pdf]

Supplementary Information (SI) for EES Solar.
This journal is © The Royal Society of Chemistry 2026

Supporting Information

Ambient Operando Self-Healing in Tin Perovskite Solar Cells

Miriam Minguez-Avellan[1], Omar E. Solis[1], Noemi Farinós-Navajas[1], Pablo F. Betancur[1], Jorge Pascual[3], Teresa S. Ripolles[1,*], Rafael Abargues[1,*], Pablo P. Boix[2,*]

[1]Instituto de Ciencia de los Materiales- Universidad de Valencia, Catedrático José Beltrán, 2, 46980, València, Spain.

[2]Instituto de Tecnología Química, Instituto de Tecnología Química, Universitat Politècnica València-Consejo Superior de Investigaciones Científicas, Av. dels Tarongers, 46022 València, Spain.

[3]Polymat, University of the Basque Country UPV/EHU, Donostia-San Sebastian, 20018, Spain

Corresponding authors: teresa.ripolles@uv.es, rafael.abargues@uv.es, Pablo.P.Boix@itq.upv.es

Experimental section

Solutions and devices were fabricated as discussed in ref.[1]. For the benefit of the reader, we provide a quote of this section below:

Materials. Tin iodide (SnI_2, 99.999%), tin (IV) iodide (SnI_4, 99.999%), tin fluoride (SnF_2, 99%), N,N-dimethylformamide (DMF, ≥ 99.8%, anhydrous), dimethyl sulfoxide (DMSO, 99.9%, anhydrous), chlorobenzene (CB, 99.8%, anhydrous), hydroiodic acid (HI, 57%), ethylenediammonium diiodide ($EDAI_2$, ≥ 98.0%) and diethyl ether (DE, ≥ 99) were purchased from Sigma Aldrich. Formamidinium iodide (FAI, $HC(NH_2)_2I$, 99.99%), Phenethylammonium iodide (PEAI) and 4-fluoro-phenethylammonium iodide (FPEAI) were purchased from Greatcell Solar Materials. 2-thiopheneethylamine (TEA, >98%) was purchased from TCI Chemicals. Fullerene (C_{60}) and bathocuproine (BCP) were purchased from 1-Materials. PEDOT:PSS AI 4083 aqueous dispersion was purchased from Heraeus. All the purchased chemicals were used as received without any further purification.

Synthesis of TEAX (X = I, Br and Cl). Briefly, 20 mL of methanol and 1.2 mL of TEA (0.01 mol) were mixed in a 50 mL round-bottom flask at room temperature under magnetic stirring. After that, reaction mixture was purged under a nitrogen flux, and subsequently, 1 molar equivalent of HX (X = I, Br and Cl) were injected and stirred for 30 min. Finally, methanol was removed by evaporation in vacuum. TEAX powders were washed by filtration with DE three times and dried overnight at 60ºC in vacuum.

Preparation of perovskite solutions. The control perovskite precursor solution was prepared by dissolving 0.4 mmol of SnI_2 (149 mg), 0.4 mmol of FAI (68.8 mg), and 0.04 mmol of SnF_2 (6.2 mg) in 1 mL of a mixture of DMF:DMSO (4:1). The TEAX-based perovskite solution was prepared by dissolving 0.4 mmol of SnI_2 (149 mg), 0.36 mmol of FAI (61.9 mg), 0.04 mmol of SnF_2 (6.2 mg) and 0.08 mmol of TEAX (20.4, 16.6 and 13.1 mg for TEAI, TEABr and TEACl, respectively) in 1 mL of a mix solvents of DMF:DMSO (4:1 in volume).

Device fabrication. Patterned ITO substrates (2.0 x 1.5 cm^2, 6-8 ohm/sq, BIOTAIN CRYSTAL CO., LIMITED) were washed in an ultrasonic bath for 15 min per process. First, the substrates were washed in water with soap and rinsed with distilled water. Then,

the substrates were washed with ethanol, acetone and isopropanol, sequentially. After drying under nitrogen flow, the substrates were treated for 30 min with UV-0_3. The hole transport material (HTM) PEDOT:PSS was filtered with 0.45 μm PVDF filter and spin coated on top of the ITO substrate at 5000 rpm for 30s and annealed at 130ºC for 20 min in ambient conditions. After HTM deposition, the substrates were transferred into a N_2-filled glovebox for the perovskite deposition.

The perovskite solutions were filtered with 0.22 μm PTFE filter, the films were deposited using 30 μL of the precursor solution and spin coated with two steps, firstly, 1000 rpm for 10s and, secondly, 5000 rpm for 50s. After 22s of having started the second step, 150 μL of CB was coated like antisolvent and the annealing treatment was carried out at 100ºC for 20 min. The electron transport material (ETM) C_{60} (30 nm)_was thermally evaporated under a pressure of less than 10^{-6} mbar. Finally, the samples were transferred into a vacuum chamber, BCP (6 nm) and AG (100 nm) were sequentially deposited by thermal evaporation under a pressure less than 10^{-6} mbar.

Solution and device characterization.

J-V curves measurements in ambient conditions were taken with a Ossila solar simulator class AAA and automated J-V measurement system (T2003B3-G2009A1). The solar cells were measured in ambient conditions (T ~ 30ºC and 60% RH) without any encapsulation, with an active area of 0.084 cm^2 defined by a mask. The stability procedure is described as follows. An initial J-V curve was measured to determine the maximum power point voltage (V_{mpp}) (or the V_{OC} in other cases) and this voltage was applied to the solar cell for 5 min at continuous illumination and after those 5 min, another J-V curve was measured for determine, once again, the V_{mpp} (V_{OC}) and apply it for the next 5 min. The parameters of the solar cell were monitored and registered every J-V curve. The stability tests in which the solar cell was maintained at 0V between J-V curves were performed using a Gamry 1010E potentiostat/galvanostat.

The external quantum efficiency (EQE) was measured from the absolute spectral response (ASR). The ASR was measured using a Xe lamp solar simulator (Zolix 150W). An optical chopper (SCITEC INSTRUMENTS), monochromator (Omni-λ150), the Ossila test board (P2008A1) were used to connect the device. A Si calibrated photodiode (ThorLabs FDS100) was used as reference sample. ASR was measured with a lock-in amplifier (STANFORD RESEARCH SYSTEMS, SR810DSP). The monochromator and the lock-in were controlled through LabView software. EQE was calculated with the spectral response with the next equation:

$$EQE_{(\lambda)} = \frac{I_{SC_{sample}}}{I_{SC_{ref}}}\left(\frac{1240}{\lambda}\right)(ASR)$$

Where, $EQE_{(\lambda)}$ is the external quantum efficiency for each wavelength. $I_{SC_{sample}}$ and $I_{SC_{ref}}$ are the currents generated for the sample and the reference sample, respectively. λ is the wavelength and ASR is the absolute spectral response.

For the integrated J_{SC} from the EQE, next equation was used:

$$J_{SC_{(\lambda)}} = \int \left[\left(EQE_{(\lambda)}\right)\left(AM1.5G_{(\lambda)}\right)\left(\frac{\lambda}{1240}\right)\right] d\lambda$$

Where, $J_{SC_{(\lambda)}}$ is the short circuit current density, EQE is the external quantum efficiency, AM1.5G is the solar spectra irradiance, and λ is the wavelength. All parameters are as a function of λ.

UV-Vis spectra were measured with a JASCO V-770 spectrophotometer with the wavelength range from 300 nm to 1000 nm with a step size of 5 nm. Solutions for UV-Vis spectroscopy were prepared as follows: SnI_2, SnI_4, TEAI, PEAI, FPEAI and $EDAI_2$ were each dissolved in a DMF:DMSO mixture (4:1 v/v) at a concentration of 0.004M. After recording the UV-Vis spectra of the individual components, each organic salt was separately added to the SnI_4 solution in equimolar proportion, and the spectra were measured again.

Liquid-state ^{119}Sn-NMR. The spectra were all acquired on a Bruker Neo500 spectrometer with Bruker 500 MHz (11.7 T) shielded magnet with standard mouth (54mm). Solutions for NMR analysis were prepared according to the following procedure: SnI_2, SnI_4 and TEAI were each dissolved in deuterated DMSO (DMSO-d_6) at a concentration of 1M and measured. Subsequently, TEAI was added to the SnI_4 solution in an equimolar ratio, and the resulting solution was measured again.

Impedance spectroscopy was carried out using a Gamry 1010E potentiostat/galvanostat on complete solar cells under ambient conditions and an illumination intensity of 100 mW cm^{-2}. Measurements were performed under different DC bias voltages ranging from open circuit to 0 V, with a 10 mV AC perturbation ranging between 1 MHz to 0.1 Hz.

PLQY and QFLS measurements were carried out using a LuQY Pro radiative efficiency meter by Quantum Yield Berlin (QYB) using a 532 nm wavelength laser with a spot size of 1 cm^2 in an integrating sphere. The signal was compiled using QYB software. The PLQY is calculated by

$$LuQY = \eta_{ext} = \left(\frac{\int_0^\infty \Phi_{lum}(E)dE}{\int_0^\infty \Phi_{exc}(E)dE - \int_0^\infty \Phi_{exc,R}(E)dE + I_{SMU}}\right) \quad (1)$$

Where η_{ext} is the external quantum yield, Φ_{lum} is the absolute luminescence flux density, E is the photon energy, Φ_{exc} is the excitation photon flux density (laser in LuQY Pro), $\Phi_{exc,R}$ is the non-absorbed excitation photon flux density (reflected laser in LuQy Pro), I_{SMU} is the current sourced by the SMU.

In-operando PLQY measurements were conducted using the LuQY Pro setup programmed to replicate the stability testing conditions described in the previous section. The measurement protocol consisted of sequential cycles as follows: first, the laser intensity was automatically set to simulate 1 sun illumination conditions. An initial PL spectrum was recorded, immediately followed by a J-V measurement to determine the device's photovoltaic characteristics. The V_{mpp} was calculated from the J-V curve and subsequently applied to the solar cell for 5 minutes under continuous simulated 1 sun illumination. After this period, a new PL spectrum was acquired, followed by another J-V measurement to reassess device performance. The updated V_{mpp} was calculated and applied for the subsequent 5 min illumination cycle. This sequential process was repeated continuously throughout the measurement period, enabling real-time monitoring of both photoluminescence properties and photovoltaic performance under operational conditions.

The QFLS is calculated using the high energy tail fit:

$$\ln\left(\frac{\Phi_{lum}(E)h^3c^2}{2\pi E^2 a(E)}\right) = -\left(\frac{E-QFLS}{kT}\right), (2)$$

Where h is the Planck constant, c is the speed of light, a is the absorptance of the sample, k is the Boltzmann constant, T is the temperature of electron gas.

Ideality factor was extracted from QFLS vs. laser intensity sweeps also calculated with the LuQY Pro software following Caprioglio et al. work[2].

Series resistance (R_S) and shunt resistance (R_{SH}) were determined from the inverse slope near the V_{OC} and I_{SC}, respectively[3], [4], [5].

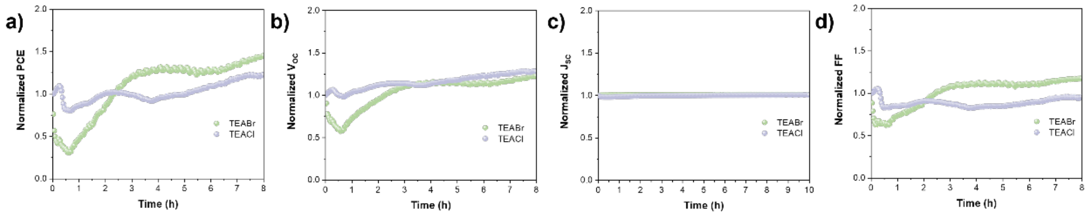

Figure S1. (a-d) Time evolution of solar cells normalized parameters of $FASnI_3$-TEABr and $FASnI_3$-TEACl solar cells with measured under operation conditions of maximum power point (MPP) and 1 sun illumination (100 mW cm^{-2}) at ambient conditions of 30ºC and 60% RH.

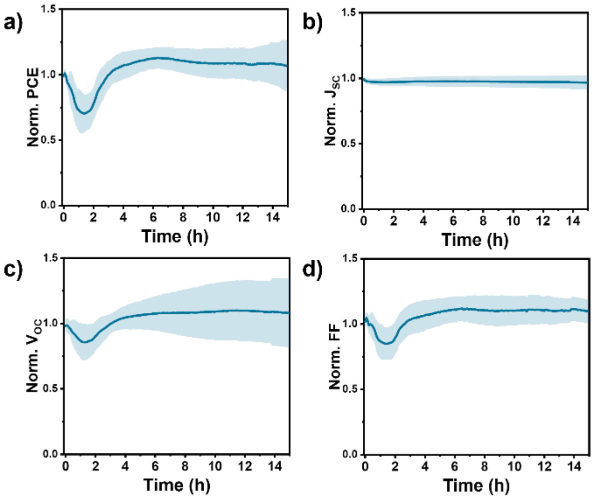

Figure S2. (a-d) Time evolution of mean normalized parameters and its standard deviation of 8 FASI-TEAI solar cells measured under operation conditions of maximum power point (MPP) and 1 sun illumination (100 mW cm-2) at ambient conditions of 30ºC and 60% RH.

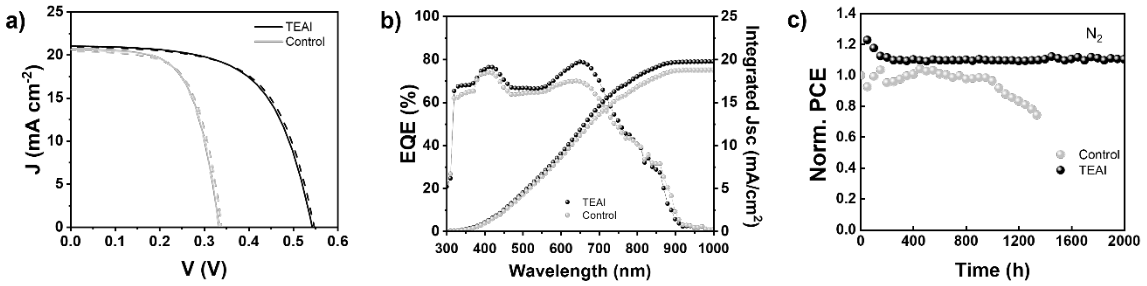

Figure S3. (a) Representative jV curves, **(b)** EQE and integrated J_{SC} and **(c)** time evolution of normalized PCE of pristine $FASnI_3$ (control) and $FASnI_3$-TEAI (TEAI) solar cells measured under operation conditions of maximum power point (mpp) and 1 sun illumination (100 mW cm^{-2}) at N_2 atmosphere.

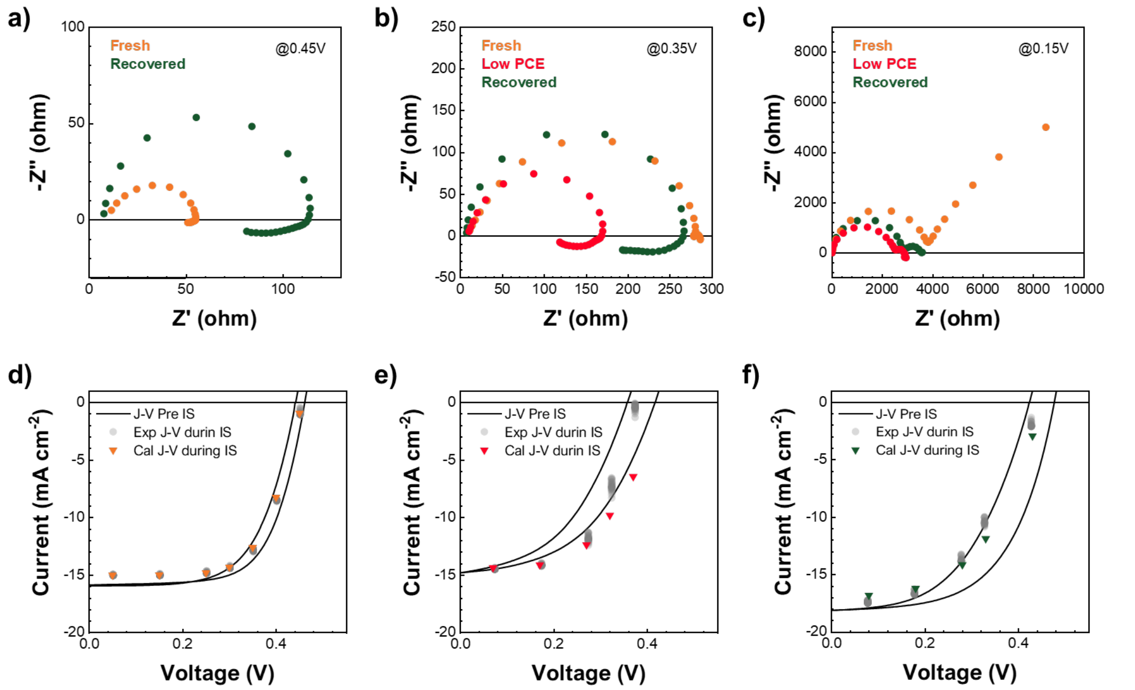

Figure S4. Nyquist plots of the device at different stages under one sun illumination at various applied voltages: **(a)** 0.45 V **(b)** 0.35 V, and **(c)** 0.15 V. Stability during IS measurements and J-V reconstruction for **(d)** fresh device, **(e)** degraded (low PCE) device, and **(f)** recovered device. Solid line represents J-V pre-IS, dots represent DC current values measured during the IS at each specific voltage bias, and triangles represent calculated current from R_{rec} values extracted from IS spectra.

In devices where charge extraction is not the limiting parameter in their performance, as is the case for all the stages analyzed in this work, the reconstruction of the J-V curve from the recombination resistance (R_{rec}) is a method that allows the identification of the R_{rec} from the IS data[6]. Using the fresh device as a reference, which shows great stability during the IS (Figure S4d), we assume the value where the Nyquist plot crosses the real axis at low frequencies to be the R_{rec} of our system. The agreement between the experimental j_{dc} and the calculated j_{dc} for each voltage indicates an accurate identification of the recombinative systems behavior. The same approach is used for the IS measurements in the device with low PCE and the recovered device (Figure S4e and f respectively), achieving a close match between the experimental and calculated data, however the volatile nature of the system at these stages is responsible for the discordance close to Voc.

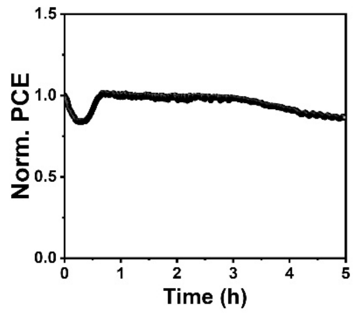

Figure S5. Time evolution of $FASnI_3$-TEAI champion solar cell normalized PCE measured under operation conditions of maximum power point (MPP) and 1 sun illumination (100 mW cm^{-2}) at ambient conditions of 30ºC and 60% RH.

Reducing activity of TEAI

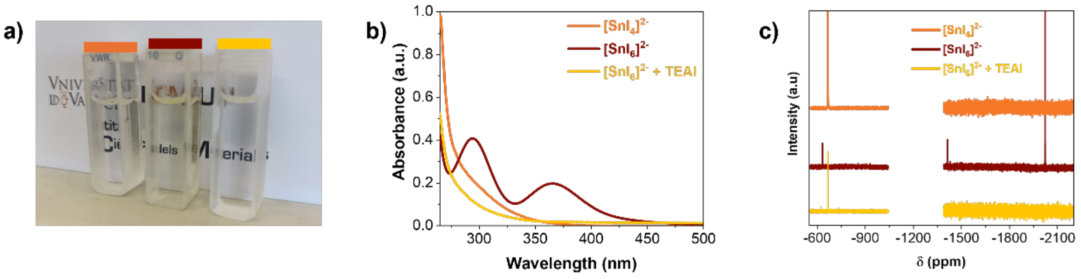

Figure S6. (a) Picture of solutions of $[SnI_4]^{2-}$ (orange), $[SnI_6]^{2-}$ (red) and $[SnI_6]^{2-}$ + TEAI (yellow) **(b)** their respective UV-vis absorption spectra and **(c)** ^{119}Sn-NMR spectra of similar solutions in $DMSO_{d6}$.

To evaluate the potential of the TEAI additive to chemically reduce Sn^{4+} to Sn^{2+}, we conducted a comparative analysis using UV-vis absorption and NMR spectroscopy on solutions of Sn(II) and Sn(IV) with I^-, and the consequent addition of TEAI to the last solution (Figure S6a). The initial Sn(II) solution forms the complex $[SnI_4]^{2-}$, and the Sn(IV) solution forms $[SnI_6]^{2-}$. In the UV-vis absorption spectra, Figure S6b, the $[SnI_6]^{2-}$ solution exhibits two characteristic absorption peaks at 290 and 365 nm. Upon addition of TEAI, this peak disappears, indicating the reduction of iodine to iodide[7] and thus suggesting that Sn^{4+} is being reduced to Sn^{2+}[8]. This reduction process is further supported by the NMR measurements. In the spectra of the $[SnI_6]^{2-}$ + TEAI solution from Figure S6c, no signal is observed in the region around 2000 ppm, where Sn^{4+} species typically resonate[9]. Furthermore, the signal near 670 ppm, overlaps with that of the $[SnI_4]^{2-}$ solution, confirming the conversion of Sn^{4+} to Sn^{2+}. These combined results provide strong evidence that the TEAI additive facilitates the in situ reduction of Sn^{4+} to Sn^{2+} in solution.

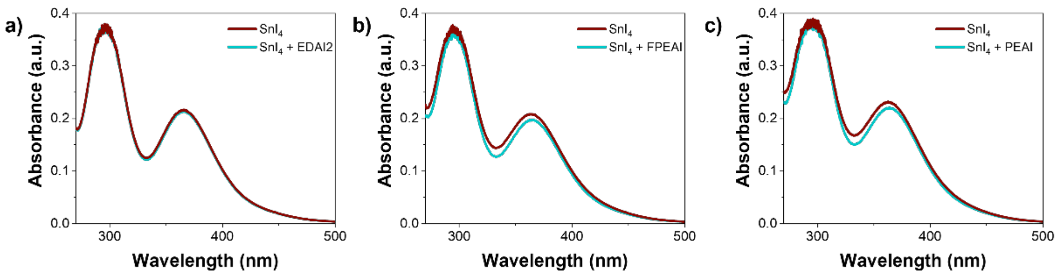

Figure S7. UV-vis absorption spectra of solutions of SnI_4 before and after the addition of **(a)** $EDAI_2$, **(b)** FPEAI and **(c)** PEAI salts.

Table S1.- Parameters of the $FASnI_3$-TEAI solar cells.

Sample	V_{OC} (V)	J_{SC} (mA cm^{-2})	FF (%)	PCE (%)
1	0.48	20.48	70.73	7.11
2	0.59	21.78	70.65	9.10
3	0.48	20.72	69.00	7.08
4	0.51	20.42	70.51	7.33
5	0.59	21.28	70.52	8.89
6	0.60	21.43	70.94	9.11

7	0.61	21.35	70.16	9.10
8	0.60	21.23	69.53	8.87
9	0.50	20.00	68.29	6.83
10	0.52	21.50	68.98	7.64
11	0.63	22.57	72.39	10.28
12	0.61	22.08	72.63	10.44
13	0.51	21.17	69.88	7.62
14	0.65	22.01	72.01	10.24
15	0.50	21.66	69.16	7.43
16	0.51	20.53	62.72	6.55
17	0.66	21.86	72.63	10.33
18	0.66	22.21	70.17	10.27
19	0.66	21.88	73.21	10.73
20	0.67	21.66	69.64	10.42
21	0.55	20.96	61.52	7.06
22	0.54	21.02	61.10	6.97
23	0.67	22.08	70.14	10.27
24	0.56	18.82	66.92	6.72
25	0.53	21.69	63.49	6.88
26	0.67	21.79	75.13	10.78
27	0.51	20.70	63.02	6.68
28	0.51	19.82	69.33	7.00
29	0.66	22.15	74.15	10.64
30	0.50	20.85	68.67	7.15
31	0.66	21.84	72.31	10.43
32	0.50	20.45	69.62	7.09
33	0.51	20.39	70.91	7.35
34	0.64	22.37	72.40	10.50
35	0.51	20.22	71.36	7.33
36	0.49	20.26	67.60	6.77
37	0.51	20.11	71.28	7.26
38	0.66	22.06	73.59	10.67
39	0.57	19.89	65.95	7.48
40	0.57	19.89	66.57	7.54
41	0.66	21.63	70.63	10.49
42	0.57	19.90	67.20	7.59
43	0.67	22.08	70.95	10.39
44	0.56	18.19	68.57	7.01
45	0.56	18.19	68.49	7.02
46	0.58	18.21	67.40	7.15
47	0.57	18.19	68.21	7.11
48	0.57	18.19	68.50	7.05
49	0.59	18.21	67.01	7.16
50	0.64	18.11	61.02	7.07

Table S2.- Parameters of the control $FASnI_3$ solar cells.

Sample	V_{OC} (V)	J_{SC} (mA cm^{-2})	FF (%)	PCE (%)
1	0.40	20.59	51.87	4.89
2	0.43	20.55	64.36	6.25
3	0.37	22.32	50.17	4.34
4	0.43	20.33	63.33	6.31
5	0.40	20.36	48.24	4.60
6	0.43	20.58	32.07	6.36
7	0.43	20.14	63.72	6.29
8	0.41	20.25	52.32	4.77
9	0.40	20.23	52.30	4.68
10	0.35	21.14	50.13	4.33
11	0.43	20.34	62.02	6.29
12	0.39	21.27	46.77	4.20
13	0.40	20.34	48.57	4.61
14	0.46	20.12	53.91	5.22
15	0.45	20.10	52.59	4.98
16	0.46	20.12	53.91	5.22
17	0.46	20.02	54.02	5.28
18	0.40	20.12	57.32	5.67
19	0.46	20.11	53.47	5.21
20	0.45	20.17	54.53	5.33
21	0.41	20.97	48.30	4.11
22	0.42	20.11	49.28	4.11
23	0.42	20.13	54.23	4.90
24	0.41	21.33	49.05	4.29
25	0.46	21.33	60.79	6.58
26	0.46	19.10	67.98	6.04
27	0.48	19.05	68.84	6.23
28	0.44	20.23	50.43	4.96
29	0.48	20.25	65.67	6.98
30	0.43	20.23	47.84	4.62
31	0.47	20.30	63.28	6.60
32	0.43	20.12	44.07	4.16
33	0.47	20.81	60.41	6.18
34	0.43	22.50	46.56	4.53
35	0.43	20.25	63.04	6.62

Table S3.- Average of the parameters of the $FASnI_3$ solar cells with different TEAX.

Sample	V_{OC} (V)	J_{SC} (mA cm^{-2})	FF (%)	PCE (%)
TEABr	0.6 ± 0.1	21.2 ± 0.4	68 ± 5	8.1 ± 0.5
TEACl	0.4 ± 0.2	20.1 ± 0.3	67 ± 5	6.3 ± 0.4

References

[1] O. E. Solis *et al.*, ‘Adjusting the Crystallization of Tin Perovskites through Thiophene Additives for Improved Photovoltaic Stability’, *ACS Energy Lett.*, vol. 9, no. 11, pp. 5288–5295, Nov. 2024, doi: 10.1021/acsenergylett.4c01875.

[2] P. Caprioglio *et al.*, ‘On the Origin of the Ideality Factor in Perovskite Solar Cells’, *Advanced Energy Materials*, vol. 10, no. 27, Jul. 2020, doi: 10.1002/aenm.202000502.

[3] D. S. H. Chan and J. C. H. Phang, ‘Analytical methods for the extraction of solar-cell single- and double-diode model parameters from I-V characteristics’, *IEEE Transactions on Electron Devices*, vol. 34, no. 2, pp. 286–293, Feb. 1987, doi: 10.1109/T-ED.1987.22920.

[4] M. Diantoro, T. Suprayogi, A. Hidayat, A. Taufiq, A. Fuad, and R. Suryana, ‘Shockley’s Equation Fit Analyses for Solar Cell Parameters from I-V Curves’, *International Journal of Photoenergy*, vol. 2018, no. 1, p. 9214820, 2018, doi: 10.1155/2018/9214820.

[5] K. Tada, ‘What Do Apparent Series and Shunt Resistances in Solar Cell Estimated by *I – V* Slope Mean? Study with Exact Analytical Expressions’, *Physica Status Solidi (a)*, vol. 215, no. 23, p. 1800448, Dec. 2018, doi: 10.1002/pssa.201800448.

[6] P. F. Betancur, O. E. Solis, R. Abargues, T. S. Ripolles, and P. P. Boix, ‘Recombination resistance identification through current–voltage curve reconstruction in perovskite solar cells’, *Phys. Chem. Chem. Phys.*, vol. 26, no. 48, pp. 29904–29912, 2024, doi: 10.1039/D4CP04143G.

[7] N. Aristidou *et al.*, ‘The Role of Oxygen in the Degradation of Methylammonium Lead Trihalide Perovskite Photoactive Layers’, *Angew Chem Int Ed Engl*, vol. 54, no. 28, pp. 8208–8212, Jul. 2015, doi: 10.1002/anie.201503153.

[8] L. Lanzetta *et al.*, ‘Degradation mechanism of hybrid tin-based perovskite solar cells and the critical role of tin (IV) iodide’, *Nat Commun*, vol. 12, no. 1, p. 2853, May 2021, doi: 10.1038/s41467-021-22864-z.

[9] J. Pascual *et al.*, ‘Fluoride Chemistry in Tin Halide Perovskites’, *Angew Chem Int Ed*, vol. 60, no. 39, pp. 21583–21591, Sep. 2021, doi: 10.1002/anie.202107599.
